# MRI for Predicting Response and 10-Year Outcome of Neoadjuvant Chemotherapy with or Without Additional Bevacizumab Treatment in HER2-Negative Breast Cancer

**DOI:** 10.3390/cancers18030393

**Published:** 2026-01-27

**Authors:** Siri Helene Bertelsen Brandal, Torgeir Mo, Anne Fangberget, Line Brennhaug Nilsen, Oliver Marcel Geier, Hilde Bjørndal, Marit Muri Holmen, Olav Engebråten, Øystein Garred, Knut Håkon Hole, Therese Seierstad

**Affiliations:** 1 Department of Breast Diagnostics, Division of Radiology and Nuclear Medicine, Oslo University Hospital, 0379 Oslo, Norway; 2 Institute of Clinical Medicine, Faculty of Medicine, University of Oslo, 0318 Oslo, Norway; torgeirmo@gmail.com (T.M.); oen@ous-hf.no (O.E.); khh@ous-hf.no (K.H.H.); 3 Department of Cancer Genetics, Institute for Cancer Research, Oslo University Hospital, 0310 Oslo, Norway; 4 Department of Oncologic Radiology, Division of Radiology and Nuclear Medicine, Oslo University Hospital, 0379 Oslo, Norway; 5 Department of Medical Physics, Division of Cancer Medicine, Oslo University Hospital, 0379 Oslo, Norway; 6 Department of Diagnostic Physics, Division of Radiology and Nuclear Medicine, Oslo University Hospital, 0379 Oslo, Norway; ogeier@ous-hf.no; 7 Department of Tumour Biology, Institute for Cancer Research, Oslo University Hospital, 0424 Oslo, Norway; 8 Department of Oncology, Division of Cancer Medicine, Oslo University Hospital, 0379 Oslo, Norway; 9 Department of Pathology, Division of Laboratory Medicine, Oslo University Hospital, 0424 Oslo, Norway; uxysga@ous-hf.no; 10 Department of Research and Development, Division of Radiology and Nuclear Medicine, Oslo University Hospital, 0424 Oslo, Norway; set@ous-hf.no

**Keywords:** breast cancer, magnetic resonance imaging, neoadjuvant therapy, bevacizumab, biomarkers

## Abstract

**Objectives**: To explore if MRI can monitor treatment and predict outcome in patients with human epidermal growth factor 2 (HER2)-negative breast cancer receiving neoadjuvant chemotherapy (NACT) with or without bevacizumab. **Methods**: Multiparametric MRI was performed at baseline and after 12 and 25 weeks of NACT. MRI assessment included tumour size, apparent diffusion coefficient (ADC) from diffusion-weighted imaging (DWI), and signal intensity–time curves and vascular volume transfer constant (K^TRANS^) from dynamic contrast-enhanced MRI (DCE). The reference standards were pathological complete response (pCR) at the time of surgery, and 10-year recurrence-free survival. Receiver operating characteristics analyses were performed to assess the predictive value of the MRI parameters. MRI findings and outcomes were compared between the treatment groups. **Results**: Seventy women were included from November 2008 to July 2012, with a median age of 49.5 years and median tumour diameter of 47 mm. Fourteen patients (20.0%) achieved pCR, while eleven (15.7%) had recurrence during the 10-year follow-up. The treatment significantly reduced tumour size, increased ADC, decreased K^TRANS^, and shifted the signal intensity–time curves towards more benign shapes. The DCE parameters changed significantly more in the bevacizumab group. In the bevacizumab group, baseline K^TRANS^ predicted pCR (Area under curve (AUC) = 0.73), but the difference in pCR-rates between the treatment groups was not significant (*p* = 0.07). Only tumour size and shrinkage at 12 weeks predicted pCR (AUC = 0.71–0.85) regardless of size measuring method. No MRI parameters predicted survival. **Conclusions**: All MRI parameters reflected treatment response, but no parameter predicted survival or benefit from adding bevacizumab to chemotherapy.

## Simple Summary

Breast cancer is a heterogeneous disease and treatment response and prognosis can be difficult to predict. Medical treatment prior to surgery is increasingly used to prevent tumour spread and improve survival for women with large breast cancers. Treatment aimed at tumour blood supply, such as bevacizumab, has been studied and some data support that it may benefit a selected subgroup of patients. There are, however, no tools for selecting these patients. In this study, we use MRI to compare tumours in patients treated with chemotherapy and patients treated with bevacizumab, in addition to chemotherapy, aiming to explore if any findings can help to predict treatment effect or survival. Patients in this study have been clinically followed for 10 years after treatment, giving valuable information relative to longtime survival.

## 1. Introduction

Neoadjuvant chemotherapy (NACT) is recommended in patients with locally advanced breast cancer to facilitate surgical treatment, and to reduce risk of recurrence and metastases [1]. NACT is also recommended for patients with high-risk T2 (2–5 cm) triple-negative and human epidermal growth factor receptor 2 (HER2)-positive breast tumours [1].

Angiogenesis is important for tumour growth and metastasis and is stimulated by vascular endothelial growth factor (VEGF). Bevacizumab, a humanized monoclonal antibody that binds VEGF [2], is used to treat several solid cancers [3] and is currently approved in Europe for treatment of metastatic breast cancer, in combination with chemotherapy [4]. Adding bevacizumab to NACT can improve pathological complete response (pCR) in HER2-negative breast cancer [5,6,7,8,9]. Different results regarding overall survival were reported [10,11].

In our neoadjuvant phase II prospective clinical trial, patients with large T2 or locally advanced HER2-negative breast cancer were randomized to NACT with or without bevacizumab [9]. The majority of patients who achieved pCR were those treated with bevacizumab combined with chemotherapy [9]. This may indicate that a subset of patients benefits from neoadjuvant bevacizumab. Bevacizumab can, however, cause serious adverse effects, and a validated method to identify patients likely to benefit from anti-angiogenic treatment is warranted.

MRI is currently the most accurate method of evaluating tumour size during NACT [12,13,14] and is recommended prior to and after NACT [15]. MRI may provide prognostic information and demonstrate treatment response by tumour extent, cellularity, and vascularization utilizing morphological, diffusion-weighted (DWI), and dynamic contrast-enhanced (DCE) images [16,17,18,19,20,21]. However, there is no consensus on which methods to use when evaluating anti-angiogenic treatment given in combination with chemotherapy. Wedam et al. already showed, in 2006, that DCE MRI parameters reflected reduced angiogenesis following treatment with bevacizumab [22].

The main aim of this study was to explore if multiparametric MRI can identify patients likely to benefit from bevacizumab treatment. A secondary aim was to assess different response parameters and their ability to predict pCR and survival.

## 2. Materials and Methods

### 2.1. Study Cohort

The current study is a radiological sub-study of the NeoAva trial, a prospective, multicenter, phase II translational clinical study designed with an explorative aim to identify potential molecular and imaging biomarkers of treatment response to neoadjuvant breast cancer treatment. The parent study was not powered to assess treatment efficacy, but to generate biomarker data to inform future hypothesis-driven research. Patients were recruited from three hospitals in Norway (St. Olavs Hospital, Ullevaal Hospital, and the Norwegian Radium Hospital). The study was approved by the institutional protocol review board, the Regional Ethics Committee (2010/335), and the Norwegian Medical Agency and is registered in the ClinicalTrials.gov database (NCT00773695). It was carried out in accordance with the Helsinki Declaration.

Patients with previously untreated, large (≥2.5 cm) HER2-negative breast carcinomas, without metastatic disease, were eligible for the study and were included between November 2008 and July 2012. Patients were stratified based on tumour size (t ≤ 5 cm, t > 5 cm) and hormone receptor status, and randomized 1:1, using block randomization, to chemotherapy with or without bevacizumab. The study cohort in this radiological part consists of patients that were treated and had MRI exams at the Norwegian Radium Hospital. All patients provided written informed consent prior to inclusion.

### 2.2. Treatment

Patients were randomized to NACT with or without bevacizumab (Avastin^®^, Roche, Basel, Switzerland). The chemotherapy consisted of four cycles of FEC100 (5-fluorouracil 600 mg/m^2^, epirubicin 100 mg/m^2^, and cyclophosphamide 600 mg/m^2^) every three weeks followed by taxane (paclitaxel/docetaxel) for 12 weeks. Bevacizumab 15 mg/kg was given every three weeks together with FEC100. For the subsequent 12 weeks, bevacizumab 15 mg/kg was given concomitantly with docetaxel 100 mg/m^2^ every three weeks, or bevacizumab 10 mg/kg was administered every two weeks in patients who received 80 mg/m^2^ paclitaxel weekly. Docetaxel 100 mg/m^2^ every three weeks and weekly paclitaxel 80 mg/m^2^ have somewhat different toxicity profiles and pharmacodynamics, but their overall antitumor efficacy over a 12-week period is considered clinically comparable. Docetaxel provides higher peak dose, whereas weekly paclitaxel offers continuous exposure with similar cumulative effect. The bevacizumab doses in the two taxane regimens were adjusted (15 mg/kg every three weeks with docetaxel or 10 mg/kg every two weeks with weekly paclitaxel) to provide similar cumulative anti-vascular effect. Thus, despite differences in administration intervals, dose, and drugs (docetaxel/paclitaxel), the expected biological tumour effect is considered comparable.

### 2.3. MRI Acquisition

**Figure 1:**
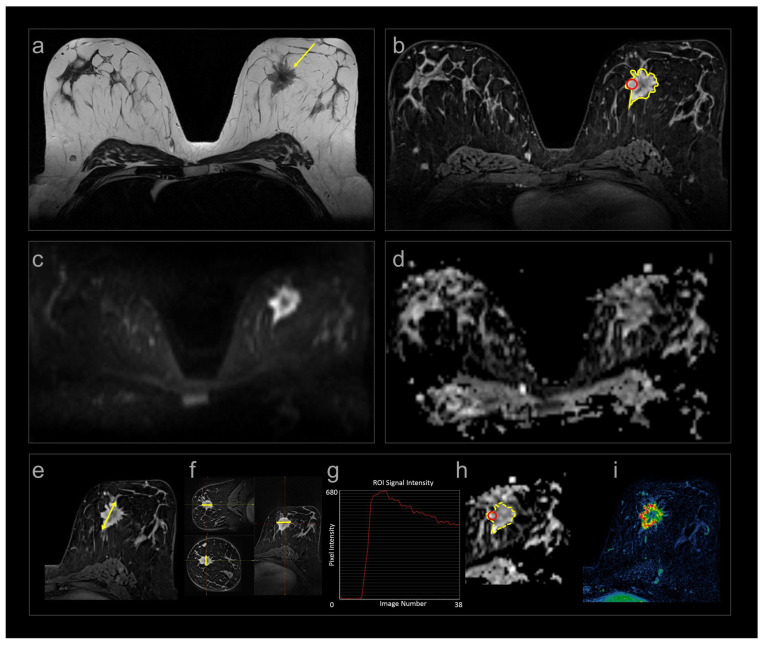
Multiparametric MRI (**a**–**d**) and measurements (**e**–**i**) in one patient at baseline. (**a**) Axial SE T2W image showing a spiculated tumour medially in the left breast (arrow); (**b**) corresponding DCE image with contrast enhancement in the tumour; (**c**) DWI b800; and (**d**) ADC maps of the same tumour. Treatment response was assessed by (**e**) longest diameter (yellow line); (**f**) calculated volume using the three perpendicular diameters (yellow lines) and the ellipsoid formula; (**b**) segmented volume (yellow delineation ); (**g**) signal intensity–time curves from focal area (red circle in (**b**)); (**h**) ADC values from focal area (red circle) and segmented volume (yellow delineation); and (**i**) quantitative volume transfer constant K^TRANS^.

The patients had multiparametric MRI at baseline and at 12 and 25 weeks after onset of NACT. The exams were all performed in prone position on a 1.5 T Siemens Magnetom ESPREE scanner (Magnetom ESPREE, Siemens, Erlangen, Germany) with a dedicated phased-array bilateral breast coil (CP Breast array coil, Siemens, Erlangen, Germany). The MRI protocol complies with the current technical EUSOBI recommendations [23] and consisted of T1W and T2W spin-echo sequences, DWI, and DCE (Figure 1, Table 1). The contrast agent Gadovist (Bayer Pharma AG, Leverkusen, Germany) 0.08 mmol/kg bodyweight was administered intravenously by a power injector, flowrate 3 mL/s. DCE was acquired using a research sequence with k-space weighted image contrast (KWIC) reconstruction [24]. The KWIC reconstruction enabled a combination of high spatial resolution for assessment of tumour extent and signal intensity–time curves, and high temporal resolution (14 s) for pharmacokinetic modelling (Supplementary Figure S1). The vascular volume transfer constant K^TRANS^, derived from DCE, was calculated with the extended Tofts model [25] as implemented in the MRI analysis suite nICE (Nordic NeuroLab, Bergen, Norway) using in-house for image pre-processing procedures written in Python (version 3.9; Numpy 1.21, OpenCV 4.3) as described in [26]. Apparent diffusion coefficient (ADC) maps were calculated by the scanner software using a mono-exponential approach with equal weighting of all five b-values.

Tumour size, ADC, and DCE curve types were included in our study because they represent established clinical tools routinely used to assess tumour extent, cellularity, and enhancement pattern in breast MRI, whereas K^TRANS^ is not a routine clinical parameter but was incorporated as a research biomarker to quantify tumour vascularity/angiogenesis, particularly relevant in the context of bevacizumab treatment.

### 2.4. MRI Reading

All exams were prospectively read by two of three radiologists (AF, MMH, HB), all with at least five years of breast MRI experience and blinded to the treatment assigned. The prospective readings included assessment of tumour size, ADC, and signal intensity–time curves. DCE images were used for size measurement and to generate signal intensity–time curves. Tumour size was measured in three dimensions: the longest diameter in the axial plane (d_axial_), the corresponding orthogonal diameter (d_ortho_), and the craniocaudal diameter (d_cc_), determined on multiplanar reconstructed images. In addition, they recorded the longest diameter in any plane (d_longest_) [27]. Tumour volume (V_calc_) was calculated using the ellipsoid formula:

$$V_{calc}=\frac{4}{3}\pi \cdot \frac{d_{axial}}{2}\cdot \frac{d_{ortho}}{2}\cdot \frac{d_{cc}}{2}$$

Lesions too small to measure were assigned a default length of 5 mm if they were still visible [27]. Median focal ADC (ADC_focal_) was obtained from a region of interest placed within the solid part of the tumour, avoiding tumour borders, areas of necrosis, and artefacts. DCE and DWI b800 images were used to guide placement of the region of interest. The signal intensity–time curves were obtained from areas of early enhancement (90 s), placed by the radiologists, and the most “malignant” curve type was assigned. They were classified as type I: persistently enhancing, type II: plateau, and type III: wash-out as described by Kuhl et al. [28].

In addition to the prospective assessments, semi-automatically segmented tumour volumes from DCE were obtained retrospectively to calculate volumes (V_seg_) and K^TRANS^ [26]. Furthermore, tumour volumes segmented from the DWI b800 images were used to calculate median ADC (ADC_seg_) [29]. An experienced breast radiologist (S.H.B.B.) reviewed the semi-automatically acquired delineations and corrected them to exclude necrosis and ensure that they only contained tumour tissue.

### 2.5. Histopathological Analysis and Clinical Long-Term Outcome

Treatment response was assessed by histopathological examination of the resected specimen. Complete response was reported when there was no remaining invasive carcinoma in either the breast or in the axillary lymph nodes. After surgery, patients were followed clinically for 10 years. Time of breast cancer recurrence, death, or last follow-up were registered. All recurrences were histologically verified.

### 2.6. Statistics

Statistical analyses were performed using SPSS v26.0 and GraphPad Prism v10.2.0. The MR imaging features analyzed were tumour size (d_axial_, d_longest_, V_calc_, V_seg_), cellularity (ADC), and vascularization (DCE curve type, K^TRANS^). Normality of continuous variables was assessed using the Shapiro–Wilk test. Normally distributed data were compared using the independent samples *t*-test while non-normally distributed or ordinal data were compared using the two-sided Mann–Whitney U test. Paired changes in MRI parameters between time points and between treatment groups were evaluated with the Wilcoxon signed-rank test. Categorical variables were compared using Pearson’s chi square test, or Fisher’s exact test when expected cell counts were <5.

Predictive performance for pCR and survival outcomes was assessed using receiver operating characteristic (ROC) curves and calculating area under the curve (AUC). Recurrence-free survival was defined as the time from baseline MRI to documented recurrence. Breast cancer-specific survival was defined as the time from baseline MRI to death from breast cancer or last follow-up. Survival distributions were estimated using Kaplan–Meier analyses, with group comparisons performed using the logrank test. The significance level was set at 5%. Because HR+/HER2− and triple-negative tumours differ in biology and treatment response, all analyses were performed both for the entire cohort and separately for the HR+/HER2− subtype, which constituted the majority of the tumours.

## 3. Results

The study included 70 women. The median age was 49.5 years. There were no differences in patients or tumour characteristics between the treatment groups at baseline (Table 2).

**Figure 2:**
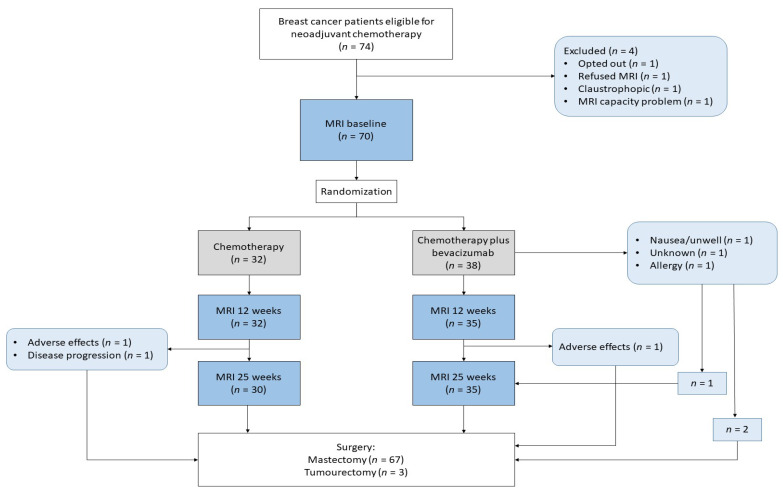
Flow chart of the study.

All patients had MRI at baseline, 67 had MRI at 12 weeks, and 65 had MRI at 25 weeks. Sixty-four had all three exams (Figure 2). All patients underwent surgery, 95.7% had a mastectomy, and all had axillary dissection. An overview of MRI data included at different time points is provided in Supplementary Table S1.

### 3.1. Monitoring Treatment Response

**Figure 3:**
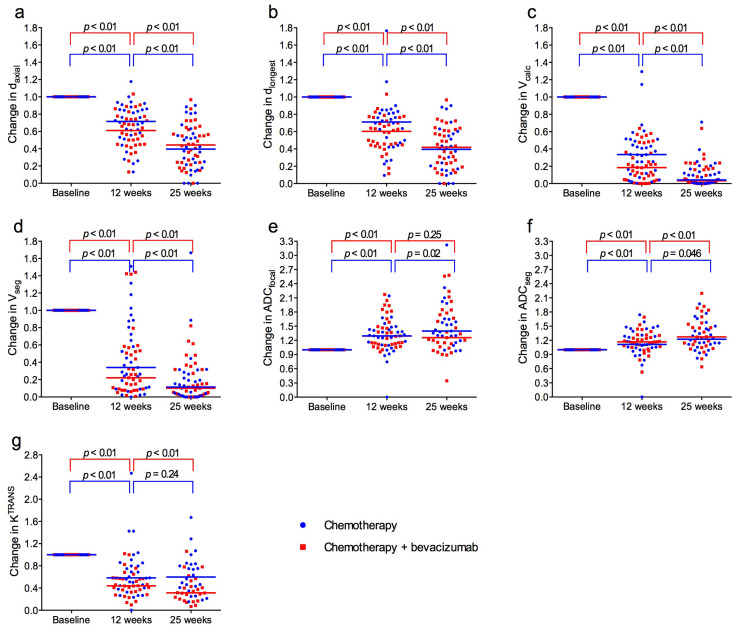
Normalized tumour size, apparent diffusion coefficient (ADC) and Volume transfer constant (K^TRANS^) at baseline and after 12 and 25 weeks for patients treated with neoadjuvant chemotherapy with (red) or without (blue) bevacizumab. (**a**) Longest axial diameter (d_axial_). (**b**) Longest diameter any plane (d_longest_). (**c**) Volume calculated from the ellipsoid formula (V_calc_). (**d**) Volume obtained by segmentation (V_seg_). (**e**) Apparent diffusion coefficient (ADC) from measuring region of interest (ADC_focal_). (**f**) ADC obtained by segmentation (ADC_seg_). (**g**) K^TRANS^. Horizontal lines represent median values in each group.

Median tumour size at baseline was 41 mm for d_axial_, 47 mm for d_longest_, 20.9 cm^3^ for V_calc_, and 10.4 cm^3^ for V_seg_. During treatment there was a significant reduction in tumour size between the three time points for all measuring methods (Figure 3a–d). The mean of the size reduction for individual patients from baseline to 12 weeks was 37% for d_axial_, 32% for d_longest_, 75% for V_calc_, and 74% for V_seg_. From baseline to 25 weeks, size reductions were 58% for d_axial_, 58% for d_longest_, 96% for V_calc_, and 90% for V_seg_. There were no significant differences between the treatment groups (Supplementary Table S2).

Median ADC_focal_ and ADC_seg_ at baseline were 1.07 × 10^−3^ mm^2^/s and 1.05 × 10^−3^ mm^2^/s. From baseline to 12 weeks, and from baseline to 25 weeks, ADC increased significantly for both measuring methods (Figure 3e,f). There were no significant differences between the treatment groups (Supplementary Table S2).

Median K^TRANS^ at baseline was 0.127 min^−1^ and there was no difference between the treatment groups. During treatment, K^TRANS^ decreased significantly in both groups, most pronounced from baseline to 12 weeks, and notably more in the bevacizumab group (Figure 3g). At 12 weeks and 25 weeks, K^TRANS^ was significantly lower in the bevacizumab group compared to the chemotherapy-only group (*p* = 0.029 and *p* = 0.017).

**Figure 4:**
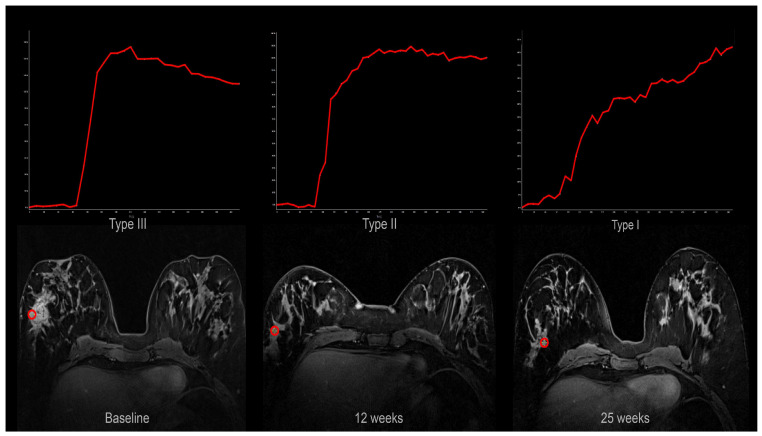
Signal intensity–time curves from one patient at baseline (type III; wash-out), 12 weeks (type II; plateau), and 25 weeks (type I; persistent). The curves were obtained from the region of interests (red circle) shown in the corresponding axial DCE MRI images.

Signal intensity–time curves shifted towards more “benign” types during treatment (Figure 4).

**Figure 5:**
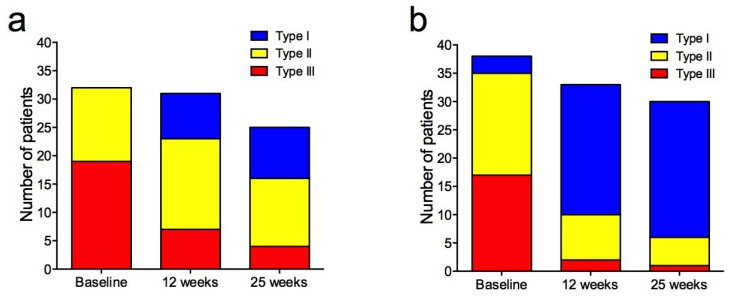
Signal intensity–time curves from dynamic contrast-enhanced MRI in patients treated with neoadjuvant chemotherapy-only (**a**) and chemotherapy + bevacizumab (**b**) at baseline, 12 weeks, and 25 weeks. Type I = persistently enhancing (blue), type II = plateau (yellow), type III = wash-out (red).

The changes were more pronounced in the bevacizumab group (Figure 5), and the distribution of curves was significantly different between the treatment groups at 12 weeks (*p* = 0.002) and 25 weeks (*p* = 0.01) (Supplementary Table S3).

### 3.2. Histopathology

Fourteen of the seventy patients achieved pCR (20%), 3/32 (9.4%) in the chemotherapy-only group, and 11/38 (28.9%) in the chemotherapy + bevacizumab group. This difference was not significant (*p* = 0.07).

### 3.3. Hormone Receptor

In our cohort, 84.3% of tumours were HR+/HER2− and 15.7% were triple-negative. Of the 59 patients with an HR+/HER2− tumour, 10 achieved pCR and 8 had recurrence. Of the 11 HR−/HER2− patients, four achieved pCR and three had recurrence. The differences between the two groups were not statistically significant.

### 3.4. Prediction of Pathological Complete Response

**Figure 6:**
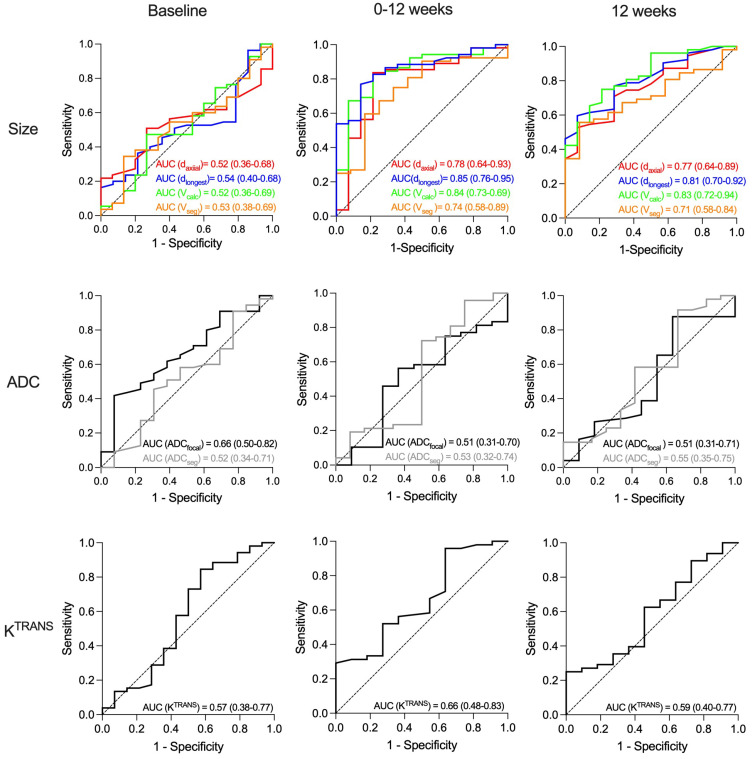
Receiver operating characteristic (ROC) curves for prediction of pathological complete response (pCR) after neoadjuvant treatment of large T2 (≥2.5 cm) and locally advanced HER2-negative breast cancer. (**Upper row**): Area under curve (AUC) for the four tumour size measurements: longest axial diameter (d_axial_), longest diameter any plane (d_longest_), calculated volume (V_calc_), and semi-automatically segmented volume (V_seg_). (**Middel row**): AUC for apparent diffusion coefficient for focal area (ADC_focal_) and segmented tumour volume (ADC_seg_). (**Lower row**): The quantitative volume transfer constant K^TRANS^ for segmented tumour volume. The three columns show the results from baseline (left), change from baseline to 12 weeks (middle) and at 12 weeks (right). The dashed line represents the diagonal reference line (AUC = 0.50).

In the study cohort as a whole, no baseline MRI parameters predicted pCR (Figure 5). At 12 and 25 weeks, only tumour shrinkage and residual tumour size predicted pCR, irrespective of measuring method (Figure 6, Supplementary Figure S2). AUC ranged from 0.71 to 0.85, without statistically significant differences between the methods (Figure 6). No ADC or K^TRANS^ values at 12 and 25 weeks or changes between time points predicted pCR. However, when assessing the chemotherapy + bevacizumab group alone, baseline K^TRANS^ predicted pCR (AUC = 0.73, 95% CI = 0.55–0.92).

### 3.5. Long-Term Outcome

**Figure 7:**
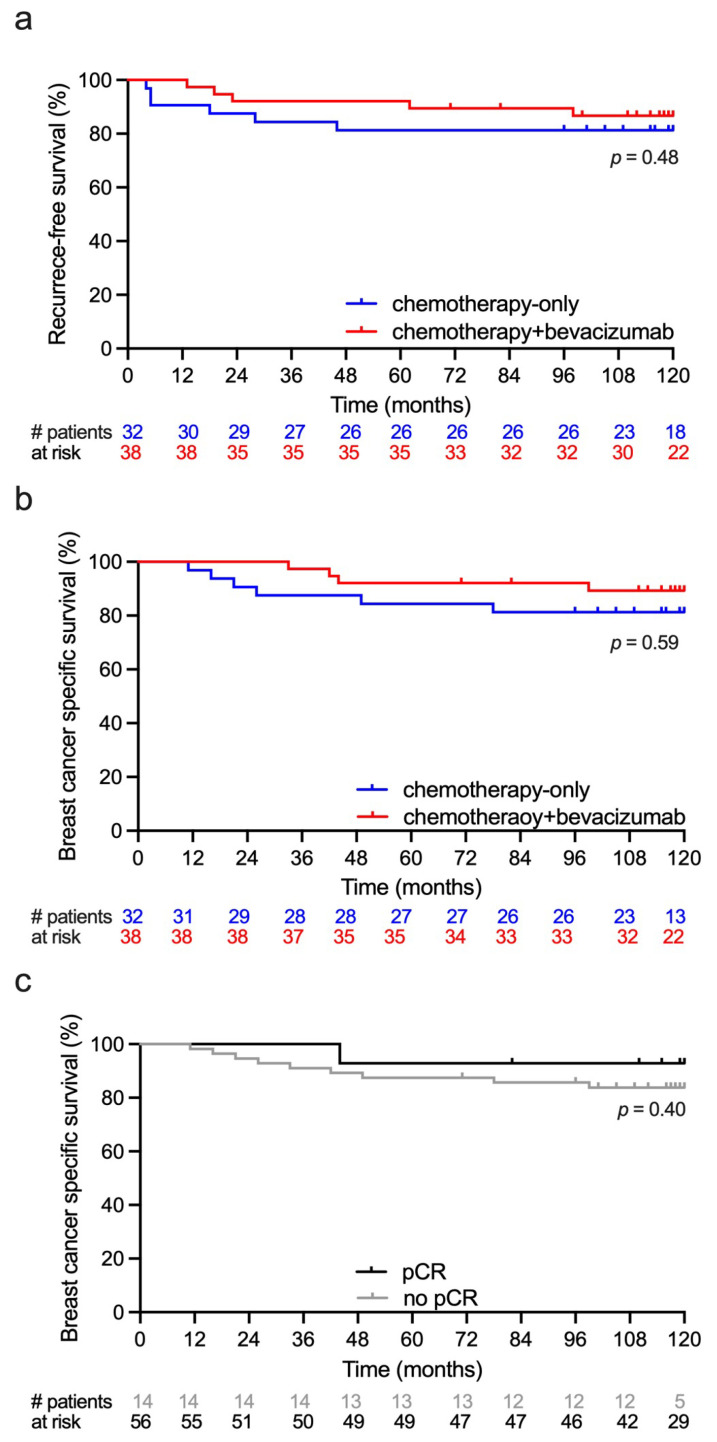
Descriptive survival curves following neoadjuvant treatment of large T2 and locally advanced HER2-negative breast cancer. Event numbers were low and results must be interpreted with caution. Red lines show survival for patients treated with chemotherapy + bevacizumab. Blue lines show survival for patients treated with chemotherapy-only. (**a**) Recurrence-free survival. (**b**) Breast cancer-specific survival. (**c**) Breast cancer-specific survival for patients achieving pathological complete response (pCR) and no pCR.

The median follow-up time was 119.5 months (range 11–120). There were eleven recurrences in the study period, six in the chemotherapy-only group, and five in the chemotherapy+ bevacizumab group. Median time from baseline MRI to recurrence was 19 months (range 4–98) and median time from recurrence to death was 21 months (range 3–37). All 11 recurrences were distant metastases and 10 died within the 10-year follow-up period. Two patients died of other primary cancers. Recurrence-free survival and breast cancer-specific survival are shown in Figure 7a,b. Breast cancer specific survival at 5 and 10 years was 85% and 80% in the chemotherapy-only group and 90% and 85% in the chemotherapy + bevacizumab group. There was no difference in survival between the treatment groups or between the pCR and non-pCR groups (Figure 7c).

### 3.6. Prediction of Long-Term Outcome

No MRI parameters or change in these during the treatment predicted breast cancer recurrence (Supplementary Figure S3). Results from separate analysis of the HR+/HER2− cohort did not differ from analysis that included the HR+/HER2− cohort.

## 4. Discussion

In this prospective study, we have explored to what extent multiparametric MRI can be used to monitor treatment response and predict pCR and outcome. The patients had HER2-negative breast cancer and were randomized to standard NACT with or without the addition of bevacizumab. We found that all size and functional MRI parameters changed significantly from baseline to 12 weeks and reflected treatment response. The decrease in K^TRANS^ and shift in signal intensity–time curve shapes were significantly larger in the bevacizumab group, indicating the anti-angiogenic effect. Of note, baseline K TRANS predicted pCR in the bevacizumab group. No MRI parameters predicted 10-year survival.

Tumour size at baseline did not correlate with outcomes. This is in line with most other studies that enrolled locally advanced cancers [30]. All tumour size measurements showed treatment response and predicted pCR irrespective of measuring method (Figure 6). A correlation between tumour shrinkage and pCR is well known [31,32,33,34]. Volumetric measurements were found to be superior to diameter in some studies [31,35], but not in others [32]. Thus, current data supports that any size measurements can be used to predict treatment response. In our cohort, tumour shrinkage predicted pCR, but did not translate into improved survival, as opposed to the ACRIN 6657-study and Partridge et al. that reported a correlation between volume shrinkage and recurrence-free survival [19,31]. The fact that our cohort consisted mainly of HR+/HER2− tumours that are known to have a weak association between pCR and outcome may have contributed to this result [20,36,37].

We found that ADC increased significantly after onset of treatment, but neither baseline ADC nor change during treatment predicted pCR or survival. This is in line with the meta-analyses from Surov et al. in 2020 [38]. However, two years later, Surov et al. reported retrospective data from a 4-centre study showing that pretreatment ADC was associated with pCR after NACT but discussed that the values overlapped in a relevant matter [39]. The ACRIN 6698-study reported that increase in ADC was moderately predictive of pCR at 12 weeks for HR+/HER2− tumours and post-treatment for both HR+/HER2− and triple-negative tumours [40]. However, HR+/HER2− tumours demonstrated the lowest pCR-rates, which may explain why we, in our smaller cohort, could not find this association. Another possible explanation is that the ACRIN study comprised a variety of treatment regimens. Hottat et al. also reported in 2023 that an increase in ADC after treatment was correlated to pCR [41]. However, they measured ADC in the same region as the baseline examination; despite that, all patients with pCR were reported to have no residual tumour at DWI after NACT (week 25). Thus, the ADC measured after NACT may not reflect tumour tissue [42]. Therefore, measuring ADC after four cycles may not be the optimal time point for response evaluation. Bedair et al. reported an increase in ADC after only three weeks correlated with pCR [43]. Interestingly, Perreira et al. found that ADC already increased after one cycle of NACT, before tumour shrinkage had occurred, and that the ADC increase correlated with pCR [44].

In our study, the signal intensity–time curves changed significantly towards more benign curve shapes after the onset of treatment. The most marked change occurred between baseline and 12 weeks and was most pronounced in the bevacizumab group. Limited data exist regarding the change in shape of the signal intensity–time curves and treatment response [45,46]. We have only found one study reporting that changes in curve shape are significantly correlated to clinical and pathological response [46].

We found that K^TRANS^ decreased significantly after the onset of treatment. However, there were no associations to pCR or survival in the cohort as a whole. Liang et al. also reported that pretreatment K^TRANS^ did not correlate to pCR, but they found that low values after treatment correlated to pCR [47]. We found no such correlation after treatment, but similar to the measurement of ADC, after substantial tumour shrinkage has occurred, K^TRANS^ may not solely reflect tumour tissue, but tumour intermixed with reparative tissue.

In the bevacizumab group, a high K^TRANS^ at baseline predicted pCR. Interestingly, in the baseline tumour biopsies from our patients Krüger et al. found that high microvessel density predicted pCR [48]. This supports that patients with highly vascularised tumours are more likely to achieve pCR when given bevacizumab. However, the difference in pCR-rates between the two treatment groups (3/32 in the chemotherapy-only group versus 11/38 in the chemotherapy + bevacizumab group) was close to but did not reach statistical significance.

We hoped that this study would reveal MRI parameters that could identify patients likely to benefit from anti-angiogenic treatment. Although patients who received anti-angiogenic treatment had a profound vascular shut-down in the tumours, this did not translate into pCR or survival benefit. The results from this study confirm that tumour shrinkage measured by MRI is a reliable method to predict pCR. However, in line with other studies, we could not show that pCR correlated to long-term survival. Logically, one would assume that pCR would translate into improved survival, but the generally good treatment results, with few recurrences, probably requires a larger number of patients to reach statistical significance. Furthermore, the correlation between pCR and survival is known to be weaker for HR+/HER2− patients [20,36,37], who constituted the majority of patients in our cohort. In our study, all size measuring methods, from a single axial diameter to advanced multiplanar segmentation, yielded comparable and significant results, reflecting treatment response. Thus, measurement of the longest tumour diameter is likely adequate in clinical practice.

The functional MRI parameters ADC and K^TRANS^ also demonstrated treatment response but did not predict pCR or survival. The only functional MRI parameter that correlated with pCR was high K^TRANS^ at baseline among the patients who received bevacizumab. However, the limited sensitivity and specificity and lack of correlation to long-term outcomes questions the clinical usefulness of baseline K^TRANS^. Functional measurements earlier in the treatment course have been reported to be predictive. Perreira et al. demonstrated that an increase in ADC preceded tumour shrinkage in patients achieving pCR. Thus, if it is clinically relevant to assess early treatment response, after one cycle of NACT, a simplified MRI using DWI without contrast agent could be an option.

As only the size measures predicted pCR, multivariable modelling was not pursued. Including variables with no evidence of effect would only have increased model instability without improving explanatory value.

The main limitation of our study is the sample size. Although it is not small compared to other studies, the number of events is low, and the cohort was divided into two different treatment groups. Our study also has strengths: Although the MRI examinations were performed between 2008 and 2012, the acquisition protocol adheres to technical standards that remain valid in current clinical practice [15]. The temporal resolution of the DCE-MRI sequence is sufficient for contemporary pharmacokinetic modelling approaches, and the diffusion-weighted imaging parameters align with current guideline recommendations. Thus, despite the age of the dataset, the functional MRI metrics extracted in this study preserve their technical relevance and can still inform today’s imaging practice. However, it should be noted that more recent scanners may provide higher signal-to-noise ratios and improved spatial resolution, which could further refine these measurements. We meticulously measured tumour size, cellularity (DWI), and vascularity (DCE), and we have a 10-year follow-up.

## 5. Conclusions

In our cohort of HER2-negative large T2 tumours and locally advanced breast cancer, both morphological and functional MRI parameters showed treatment response to neoadjuvant treatment. No MRI parameters could identify patients likely to benefit from bevacizumab. All measurements of tumour size and tumour shrinkage at twelve weeks predicted pCR. No MRI parameters predicted survival.

## Figures and Tables

**Table 1:** Magnetic resonance imaging (MRI) protocol and sequence parameters.

Acquisition Parameters	T1W	T2W	T1W DCE	DWI
Pulse sequence	2D TSE	2D TSE	3D SPGR	SE EPI
Acquisition plane	Sagittal	Transversal	Axial	Axial
Echo time (ms)	9.4	80	2.59	69
Repetition time (ms)	666	4570	5.46	5200
Flip angle (°)	150	150	15	90
Slice thickness (mm)	4	3	1.5	3
Slice gap (mm)	1	0	0	1.5
Number of excitations	1	0.66	1	6
Inplane resolution (mm)	0.43 × 0.43	0.33 × 0.33	1.00 × 1.00	2.57 × 2.57
Echo train	5	17		
Bandwidth (Hz/pixel)	220	220	460	1116
FOV (mm)	220 × 220	340 × 340	320 × 320	360 × 185
Matrix size (pixel)	512 × 512	512 × 512	320 × 320	140 × 72
b-values (s/mm^2^)				0, 50, 250, 500, 800
Time resolution (min:s)			0:14/1:51	
Acquisition time (min:s)	2:23	4:32	9:25	7:02

Abbreviations: T1W = T1-weighted, T2W = T2-weighted, DCE = dynamic contrast-enhanced, DWI = diffusion-weighted imaging, SPGR = spoiled gradient-echo, TSE = turbo spin-echo, SE EPI = spin-echo echo-planar imaging, FOV = field of view.

**Table 2:** Patient and tumour characteristics at baseline.

	All	Chemotherapy-Only	Chemotherapy + Bevacizumab	*p*
Patients	*n* = 70 (100%)	*n* = 32 (45%)	*n* = 38 (55%)	
Age (years)				0.78 ^a^
Minimum	30	30	31	
Maximum	70	64	70	
Median	49.5	50.5	48.0	
Tumour diameter (mm)				0.76 ^b^
Minimum	29.0	29.0	29.0	
Maximum	92.0	92.0	76.0	
Median	47.0	47.0	46.5	
Stage				0.73 ^b^
T2	20 (28.6)	10 (31.3)	10 (26.3)	
T3	46 (65.7)	20 (62.5)	26 (68.4)	
T4	4 (5.7)	2 (6.2)	2 (5.3)	
Histopathology				0.71 ^c^
IDC	60 (85.7)	27 (84.4)	33 (86.8)	
ILC	9 (12.9)	4 (12.5)	5 (13.2)	
IMC	1 (1.4)	1 (3.1)	0 (0.0)	
Grade				0.10 ^b^
I	6 (8.6)	0 (0.0)	6 (15.8)	
II	49 (70.0)	24 (75.0)	25 (65.8)	
III	15 (21.4)	8 (25.0)	7 (18.4)	
Estrogen receptor status				1.00 ^c^
Positive	59 (84.3)	27 (84.4)	32 (84.2)	
Negative	11 (15.7)	5 (15.6)	6 (15.8)	
Nodal status				0.74 ^b^
cN0	36 (51.4)	15 (46.9)	21 (55.3)	
cN1	5 (7.2)	4 (12.5)	1 (2.6)	
pN1	29 (41.4)	13 (40.6)	16 (42.1)	

Abbreviations: IDC = invasive ductal carcinoma, ILC = invasive lobular carcinoma, IMC = invasive mucinous carcinoma. Tumour diameter is the longest in any plane. The *p*-value is for comparing the two treatment groups. ^a^ = *t*-test, ^b^ = Mann–Whitney U test, ^c^ = Fisher’s exact test.

## Data Availability

The images and data that support the findings of this study are stored in an institutional repository and are available from the corresponding author upon reasonable request. However, due to strict GDPR- and privacy laws in Norway, data sharing would be difficult. The in-house written python code used for segmentation of tumour is available from the corresponding author upon request.

## Supplementary Materials

The following supporting information can be downloaded at: https://www.mdpi.com/article/10.3390/cancers18030393/s1, Figure S1: Dynamic contrast-enhanced MRI using *k*-space weighted image contrast (KWIC) technique; Figure S2: Receiver operating characteristic (ROC) curves for prediction of pCR and breast cancer recurrence after neoadjuvant treatment at 0–25 weeks and at 25 weeks; Figure S3: Receiver operating characteristic (ROC) curves for prediction of breast cancer recurrence after neoadjuvant treatment at baseline and 0–12 week and at 12 weeks. Table S1: Overview of available MRI exams to include in the data analyses; Table S2: Tumour characteristics at baseline, 12 and 25 weeks and change between the time points, stratified and compared by treatment and pathological complete response (pCR). Table S3: Signal intensity–time curves for all patients and comparison between the two treatment groups chemotherapy-only and chemotherapy + bevacizumab.

## Abbreviations

The following abbreviations are used in this manuscript:

ADC	Apparent diffusion coefficient
ADC_focal_	Apparent diffusion coefficient in focal area
ADC_seg_	Apparent diffusion coefficient in segmented area
AUC	Area under the curve
d_axial_	Axial diameter
d_cc_	Craniocaudal diameter
d_ortho_	Orthogonal diameter
DCE	Dynamic contrast-enhanced
DWI	Diffusion-weighted imaging
FOV	Field of view
HER2	Human epidermal growth factor 2
HR	Hormone receptor
K^TRANS^	Volume transfer constant
KWIC	k-space weighted image contrast
MRI	Magnetic resonance imaging
NACT	Neoadjuvant chemotherapy
pCR	Pathological complete response
ROC	Receiver operating characteristic
SE EPI	Spin-echo echo-planar imaging
SPGR	Spoiled gradient-echo
TSE	Turbo spin-echo
V_calc_	Calculated volume
V_seg_	Segmented volume
