# Supplementary material for: MRI for Predicting Response and 10-Year Outcome of Neoadjuvant Chemotherapy with or Without Additional Bevacizumab Treatment in HER2-Negative Breast Cancer
Source: Cancers (Basel). 2026 Jan 27;18(3):393. doi: 10.3390/cancers18030393 (PMC12897187; doi:10.3390/cancers18030393)
Supplement: Supplementary file 1 [file cancers-18-00393-s001.zip › cancers-4067148-Supplementary material/Supplementary material.pdf]

Supplementary material

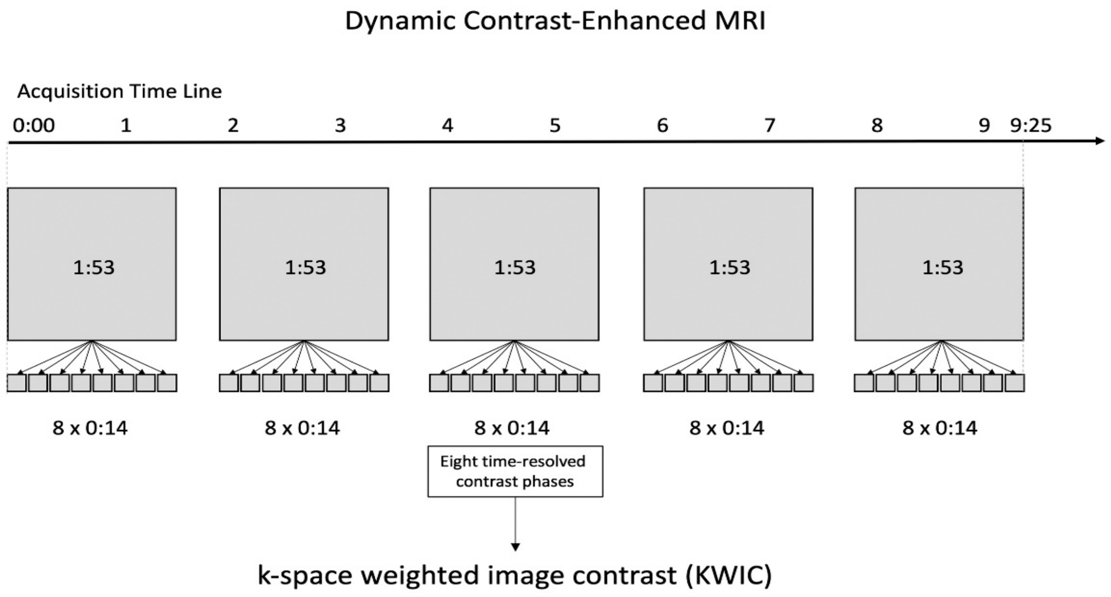

Figure S1. Dynamic contrast-enhanced MRI using *k*-space weighted image contrast (KWIC) technique. Multiple (8) acquisitions at 14 sec intervals with radial k-space filling, enabled high temporal resolution, for information on pharmacokinetics, combined with high spatial resolution for morphological detail.

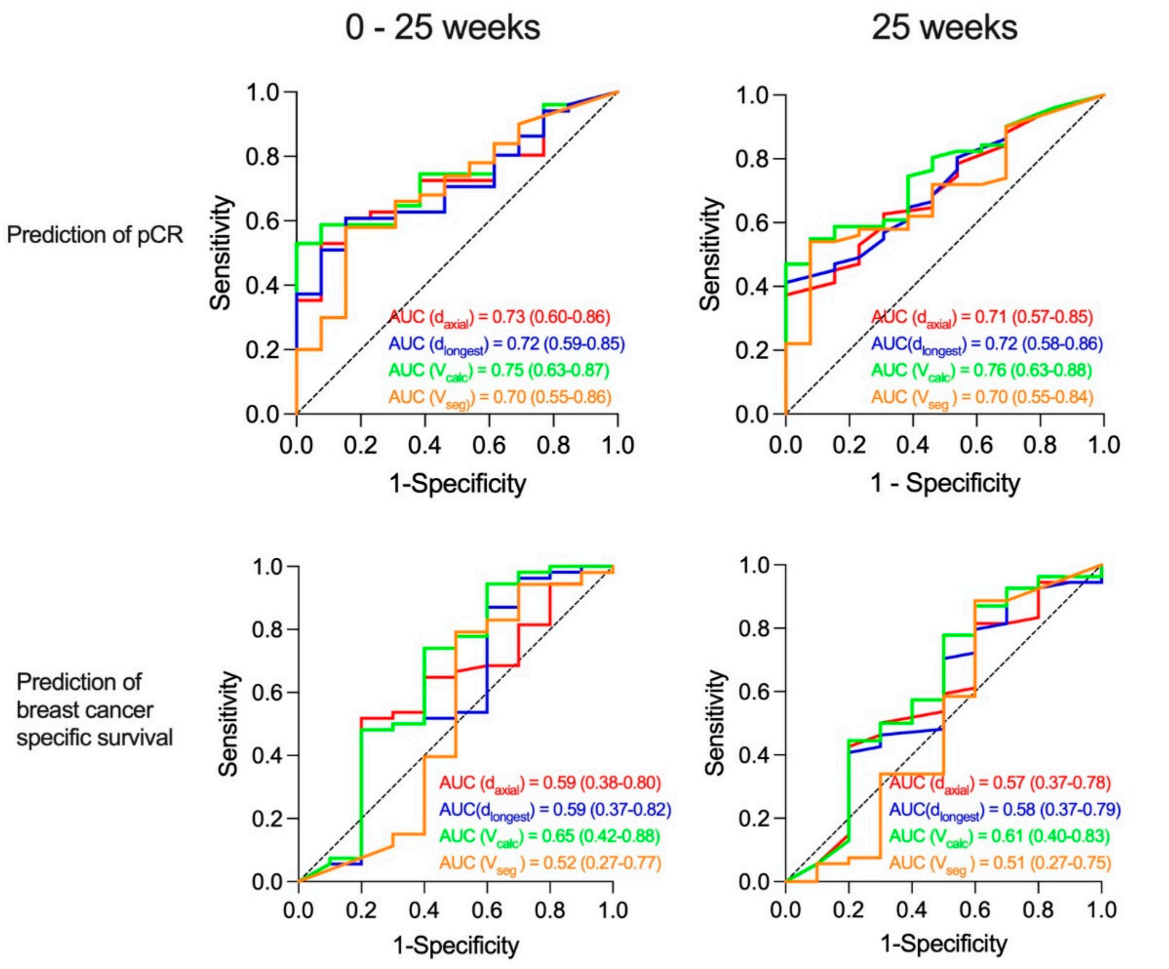

Figure S2. Receiver operating characteristic (ROC) curves for prediction of pathological complete response (pCR) (upper row) and breast cancer recurrence (lower row) after neoadjuvant treatment of large T2 and locally advanced HER2 negative breast cancer. Area under curve (AUC) for the four tumor size measurements: longest axial diameter (d_{axial}), longest diameter any plane ($d_{longest}$), calculated volume (V_{calc}) and semi-automatically segmented volume (V_{seg}). The two columns show the results for change from baseline to 25 weeks (left) and at 25 weeks (right).

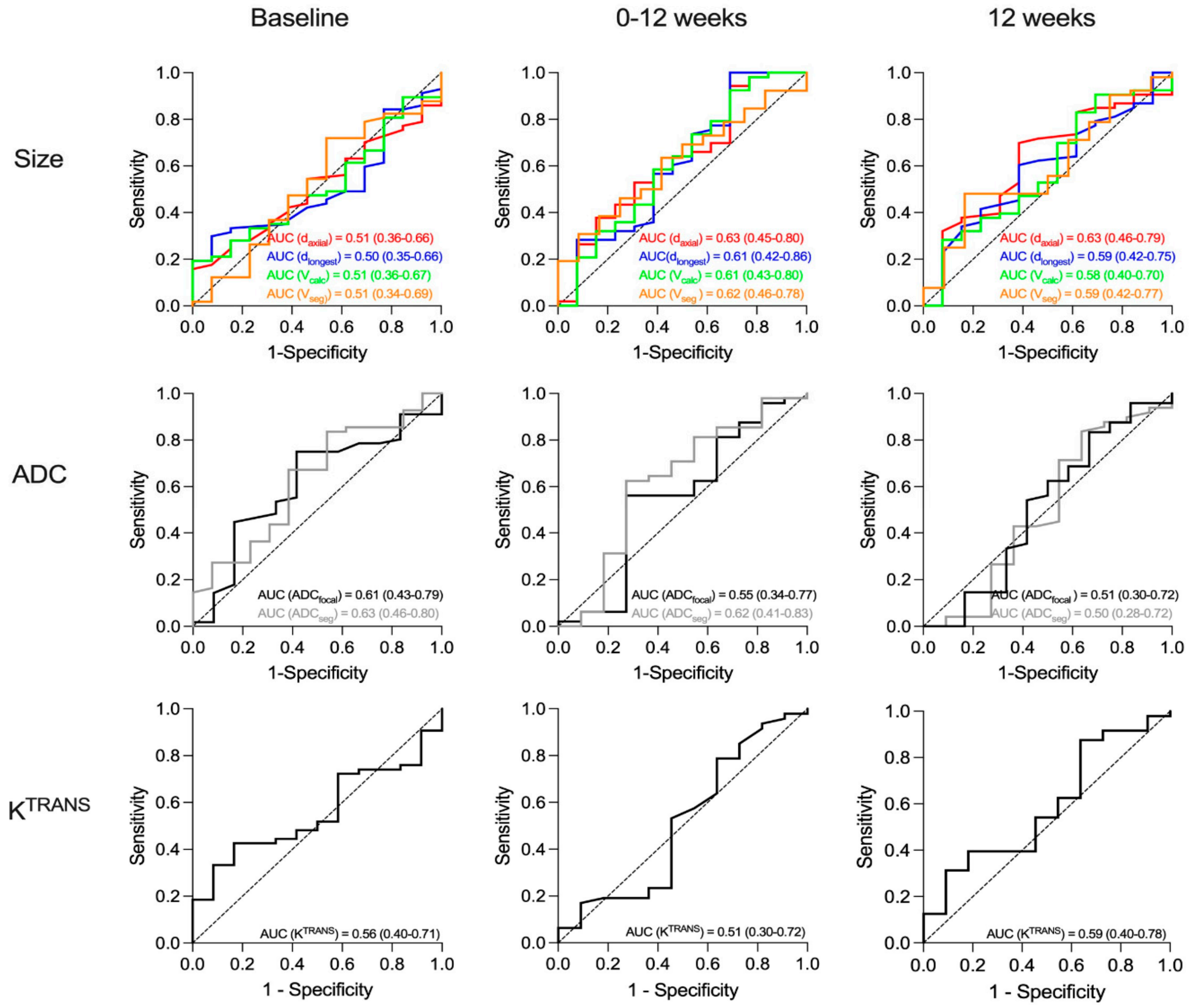

Figure S3. Receiver operating characteristic (ROC) curves for prediction of breast cancer recurrence after neoadjuvant treatment. Upper row: Area under curve (AUC) for the four tumour size measurements: longest axial diameter (d_{axial}), longest diameter any plane ($d_{longest}$), calculated volume (V_{calc}) and semi-automatically segmented volume (V_{seg}). Middle row: AUC for apparent diffusion coefficient (ADC) for focal area (ADC_{focal}) and segmented tumour volume (ADC_{seg}). Lower row: The volume transfer constant (K^{TRANS}) for segmented tumour volume. The three columns show the results for baseline (left), change from baseline to 12 week (middle) and at 12 weeks (right).

Table S1. Overview of available MRI exams include in the data analyses

	**MR baseline n = 70 (C/B)**	**MR 12 weeks n = 67 (C/B)**	**MR 25 weeks n = 65 (C/B)**
$\mathbf{V_{seg}}$	70 (32/38)	65 (32/33)<br>tumour not discernible from parenchyma (1)<br>motion artifact (1)	63 (30/33)<br>not discernible from parenchyma (2)
$\mathbf{V_{calc}}$	70 (32/38)	66 (32/34)<br>tumour not discernible from parenchyma (1)	64 (30/34)<br>not discernible from parenchyma (1)
**Diameter (d_{axial}, d_{ortho}, $d_{longest}$)**	70 (32/38)	66 (32/34)<br>tumour not discernible from parenchyma (1)	64 (30/34)<br>not discernible from parenchyma (1)
ADC_{seg}	68 (31/37)<br>fat suppression failed (2)	60 (29/31)<br>fat suppression failed (4), motion artefacts (1), not discernible from parenchyma (1), too small to measure (1)	50 (25/25)<br>fat suppression failed (1), no remaining tumour/tumour too small (14)
$\mathbf{ADC_{focal}}$	68 (31/37)<br>fat suppression failed (2)	60 (29/31)<br>fat suppression failed (4)<br>technical problem and not able to measure (1)<br>tumour not discernible from parenchyma (1)<br>tumour too small to measure (1)	54 25/29)<br>fat suppression failed (1), technical problem and not able to measure (1), tumour too small /no remaining tumour (9)
$\mathbf{K^{TRANS}}$	66 (29/37)<br>B1 normalization failed (3)<br>images missing in PACS (1)	59 (28/31)<br>missing B1 normalization images (3), not discernible from parenchyma (1), tumour too small (1), motion artefacts (1), technical error (1), image missing (1)	46 (22/24)<br>missing B1 normalization images (3), missing images (3), tumour too small/ no remaining tumour (13)
**Signal intensity-time curves**	70 (32/38)	64 (31/33)<br>technical problem (1),,not discernible from parenchyma (1)	55 (25/30)<br>tumour too small/ no remaining tumour (10)

Abbreviations: C = Chemotherapy-only, B = Chemotherapy + bevacizumab, V_{seg} = volume measured by semi - automated segmentation, V_{calc} = volume calculated from ellipsoid formula, d_{axial} = diameter in the axial plane, d_{ortho} = diameter in the axial plane, $d_{longest}$ = longest diameter any plane, ADC_{focal} = Apparent diffusion coefficient measured manually by placing ROI, ADC_{seg} = Apparent diffusion coefficient measured by semi-automated segmentation, K^{TRANS} = volume transfer constant.

Table S2. Tumour characteristics at baseline, 12 and 25 weeks and change between the time points, stratified and compared by treatment and pathological complete response (pCR).

	All	Chemotherapy + bevacizumab	Chemotherapy-only	*p* value[a]	pCR	non-pCR	*p* value[a]
**Baseline**	**median (range)**	**median (range)**	**median (range)**		**median (range)**	**median (range)**	
Longest axial diameter (cm)	4.1(1.6-9.2)	4.2(2.8-7.6)	3.0(1.6-9.2)	0.71	4.1(1.6-6.1)	4.1(2.2-9.2)	0.82
Longest diameter, any plane (cm)	4.7(2.9-9.2)	4.7(2.9-7.6)	4.7(2.9 -9.2)	0.76	4.5(3.2-6.1)	5.0(2.9-9.2)	0.51
V_{calc} (cm^3)	20.9(1.2-220.0)	21.9(5.4 -76.9)	18.7(1.2-220.0)	0.67	19.1(1.2-81.7)	20.0(4.8-220.0)	0.75
V_{seg} (cm^3)	10.4(1.1-113.7)	11.1(1.9-40.4)	9.73(1.1-113.7)	0.63	11.2(2.9-64.2)	10.2(1.1-113.7)	0.71
ADC_{focal} (x 10^{-3} mm^2/sec.)	1.07(0.70-1.63)	1.08(0.76-1.40)	1.05(0.70-1.63)	0.64	0.99(0.70-1.36)	1.08(0.76-1.63)	0.07
ADC_{seg} (x 10^{-3} mm^2/sec.)	1.05(0.67-1.52)	1.04(0.85-1.44)	1.14(0.67-1.52)	0.65	1.10(0,67-1.44)	1.04(0.84-1.52)	0.82
K^{TRANS} (min^{-1})	0.127(0.050-0.283)	0.128(0.059-0.283)	0.124(0.050-0.264)	0.61	0.139(0.060-0.283)	0.118(0.050-0.264)	0.41
**12 weeks**							
Longest axial diameter (cm)	2.6(0.5-7.2)	2.6(0.8-5.9)	2.6 (0.5-7.2)	0.57	1.0(0.8-3.1)	2.8(0.5-7.2)	0.01
Longest diameter, any plane (cm)	2.7(0.5-9.0)	2.6(0.8-5.9)	2.9 (0.5-9.0)	0.21	1.9(0.8-3.1)	3.0(0.5-9.0)	0.002
V_{calc} (cm^3)	4.0(0.1-89,7)	3.8(0.1-41.3)	4.6 (0.07-89.68)	0.47	2.0(0.1-6.5)	5.6(0.1-89.7)	<0.001
V_{seg} (cm^3)	2.8(0-43.3)	1.9(0.2-21.1)	3.6 (0-43.29)	0.42	1.9(0.1-6.2)	4.1(0.0-43.3)	0.03
ADC_{focal} (x 10^{-3} mm^2/sec.)	1.35(0.74-2.37)	1.47(0.85-2.17)	1.22 (0.74-2.37)	0.13	1.25(0.88-1.80)	1.36(0.74-2.37)	0.92
ADC_{seg} (x 10^{-3} mm^2/sec.)	1.22(0.69-1.95)	1.21(0.69-1.65)	1.23 (0.74-1.95)	0.76	1.17(0.69-1.57)	1.22(0.74-1.95)	0.61
K^{TRANS} (min^{-1})	0.059(0.018-0.264)	0.055(0.018-0.193)	0.070(0.020-0.264)	0.03	0.045(0.018-0.094)	0.059(0.033-0.264)	0.37
**Change baseline to 12 weeks**							
Reduction axial diameter (%)	32.2(-18.0-87.0)	39.0(-3.0-87.0)	28.4(-18.0-87.0)	0.12	54.8(0.0-77.0)	29.4(-18.0-87.0)	0.01
Reduction diameter, any plane (%)	36.5(-76.5-90.6)	39.7(-3.3-88.6)	28.8(-76.5-90.6)	0.10	55.9(30.8-82.2)	30.2(-76.5-90.6)	<0.001
Reduction V_{calc} (%)	74.9(-29.0-100.0)	79.4(36.0-100.0)	66.5(-29.0-100.0)	0.15	94.2(51.0-99.0)	66.5(-29.0-100.0)	<0.001
Reduction V_{seg} (%)	73.7(-51.0-100.0)	77.8(-44.0-99.0)	68.7(-51.0-100.0)	0.40	91.5(35.0-98.0)	67.5(-51.0-100.0)	0.02
Increase ADC_{focal} (x 10^{-3} mm^2/sec.)	0.33(-0.39-1.20)	0.39(-0.04-1.00)	0.30(-0.39-1.20)	0.32	0.23(0.04-0.96)	0.34(-0.39-1.20)	0.98
Increase ADC_{focal} (%)	29.4(-25.7-117.2)	29.4(-4.5-117.2)	30.0(-25.7-114.3)	0.48	25.7(3.9-117.2)	31.3(-25.7-114.4)	0.95
Increase ADC_{seg} (x 10^{-3} mm^2/sec.)	0.16(-0.68-0.71)	0.17(-0.68-0.60)	0.13(-0.40-0.71)	0.86	0.14(-0.68-0.56)	0.16(-0.40-0.71)	0.67
Increase ADC_{seg} (%)	16.8(-47.2-74.1)	18.8(-47.2-69.1)	14.0(-32.7-74.1)	0.82	18.4(-47.2-59.2)	16.8(-32.7-74.1)	0.78
Reduction K^{TRANS} (%)	49.4(-146.9-90.4)	56.3(-2.0-90.4)	41.7(-146.9-76.6)	0.03	54.2(30.9-90.4)	44.1(-146.9-86.1)	0.13
**25 weeks**							
Longest axial diameter (cm)	1.7(0.0-6.9)	1.7(0.0-4.7)	1.5(0.0-6.9)	0.59	1.1(0.0-2.2)	1.8(0.0-6.9)	0.02
Longest diameter, any plane (cm)	1.9(0.0-6.9)	1.9(0.0-4.7)	1.7(0.0-6.9)	0.78	1.1(0.0-2.2)	1.9(0.0-6.9)	0.02
V_{calc} (cm^3)	0.9(0.0-51.8)	1.0(0.0-21.9)	0.9(0.0-51.8)	0.72	0.2(0.0-1.8)	1.5(0.0-51.8)	0.004
V_{seg} (cm^3)	1.0(0.0-27.6)	1.0(0.0-15.7)	0.9(0.0-27.6)	0.67	0.4(0.0-3.7)	1.4(0.0-27.6)	0.03
ADC_{focal} (x 10^{-3} mm^2/sec.)	1.54(0.37-2.48)	1.63(0.37-2.24)	1.53(0.78-2.48)	0.46	1.68(0.92-2.24)	1.53(0.37-2.48)	0.99
ADC_{seg} (x 10^{-3} mm^2/sec.)	1.29(0.81-2.12)	1.44(0.85-2.02)	1.27(0.81-2.12)	0.73	1.29(0.81-1.66)	1.29(0.85-2.12)	0.75
K^{TRANS} (min^{-1})	0.049(0.014-0.211)	0.039(0.014-0.148)	0.057(0.026-0.211)	0.02	0.041(0.014-0.054)	0.050(0.023-0.211)	0.07
**Change baseline to 25 weeks**							
Reduction axial diameter (%)	57.9(0.0-100.0)	55.7(3.0-100.0)	60.4(10.0-100.0)	0.58	75.0(44.0-100)	50.0(3.0-100.0)	0.012

Reduction diameter, any plane (%)	57.9(3.3-100.0)	58.1(3.3-100.0)	57.9(10.0-100.0)	0.53	75.0(43.6-100.0)	51.2(3.3-100.0)	0.016
Reduction V_{calc} (%)	95.5(29.0-100.0)	95.8(36.0-100.0)	93.9(29.0-100.0)	0.54	98.7(92.0-100.0)	90.3(29.0-100.0)	0.005
Reduction V_{seg} (%)	89.9(-67.0-100.0)	89.9(18.0-100.0)	88.5(-67.0-100.0)	0.63	96.7(65.0-100.0)	85.9(-67.0-100.0)	0.024
Increase ADC_{focal} (x 10^{-3} mm^2/sec.)	0.42(-0.70-1.71)	0.35(-0.70-1.28)	0.42(-0.04-1.71)	0.44	0.37(-0.10-1.28)	0.42(-0.70-1.71)	0.69
Increase ADC_{focal} (%)	36.5(-65.4-222.1)	25.7(-65.4-157.9)	39.6(-2.6-222.1)	0.40	53.4(-9.8-156.0)	35.3(-65.4-222.1)	0.60
Increase ADC_{seg} (x 10^{-3} mm^2/sec.)	0.26(-0.52-1.10)	0.30(-0.52-1.10)	0.26(-0.22-0.98)	0.50	0.29(-0.52-0.75)	0.26(-0.24-1.10)	0.85
Increase ADC_{seg} (%)	27.2(-36.4-119.5)	27.3(-36.4-119.51)	24.3(-18.0-97.5)	0.50	31.0(-36.4-84.0)	27.2(-19.4-119.5)	0.94
Reduction K^{TRANS} (%)	59.3(-67.2-93.3)	68.7(-6.2-93.3)	40.1(-67.2-84.7)	0.007	70.9(16.4-93.3)	56.8(-67.2-85.8)	0.17

Abbreviations: pCR = pathological complete response, V_{seg}= volume measured by semi- automated segmentation, V_{calc} = volume calculated from ellipsoid formula, ADC_{focal} = Apparent diffusion coefficient measured manually by placing ROI, ADC_{seg} = Apparent diffusion coefficient measured by semi- automated segmentation, K^{TRANS} = volume transfer constant. [a] = Mann Whitney U test. Significance level p = 0.05

Table S3. Signal intensity-time curves for all patients and comparison between the two treatment groups chemotherapy-only and chemotherapy + bevacizumab

	**All**	**Chemotherapy-only**	**Chemotherapy + Bevacizumab**	p^{a}
	n (%)	*n* (%)	*n* (%)	
**Baseline**				0.15
Type I (Persistent)	3 (4.3)	0 (0.0)	3 (7.9)	
Type II (Plateau)	31 (44.3)	13 (40.6)	18 (47.4)	
Type III (Wash-out)	36 (51.4)	19 (59.4)	17 (44.7)	
Total	70 (100.0)	32 (100.0)	38 (100.0)	
**12 weeks**				0.002
Type I (Persistent)	31 (47.0)	8 (25.0)	23 (67.6)	
Type II (Plateau)	24 (36.4)	16 (50.0)	8 (23.5)	
Type III (Wash-out)	9 (13.6)	7 (21.9)	2 (5.9)	
No remaining tumour/too small to measure (missing)	2 (3.0)	1 (3.1)	1 (2.9)	
Total	66 (100.0)	32 (100.0)	34 (100.0)	
**25 weeks**				0.01
Type I (Persistent)	33 (50.8)	9 (30.0)	24 (68.6)	
Type II (Plateau)	17 (26.2)	12 (40.0)	5 (14.3)	
Type III (Wash-out)	5 (7.7)	4 (13.3)	1 (2.9)	
No remaining tumour/too small to measure (missing)	10 (15.4)	5 (16.7)	5 (14.3)	
Total	65 (100.0)	30 (100.0)	35 (100.0)	

[a] = Mann Whitney U test. Significance level $p = 0.05$
